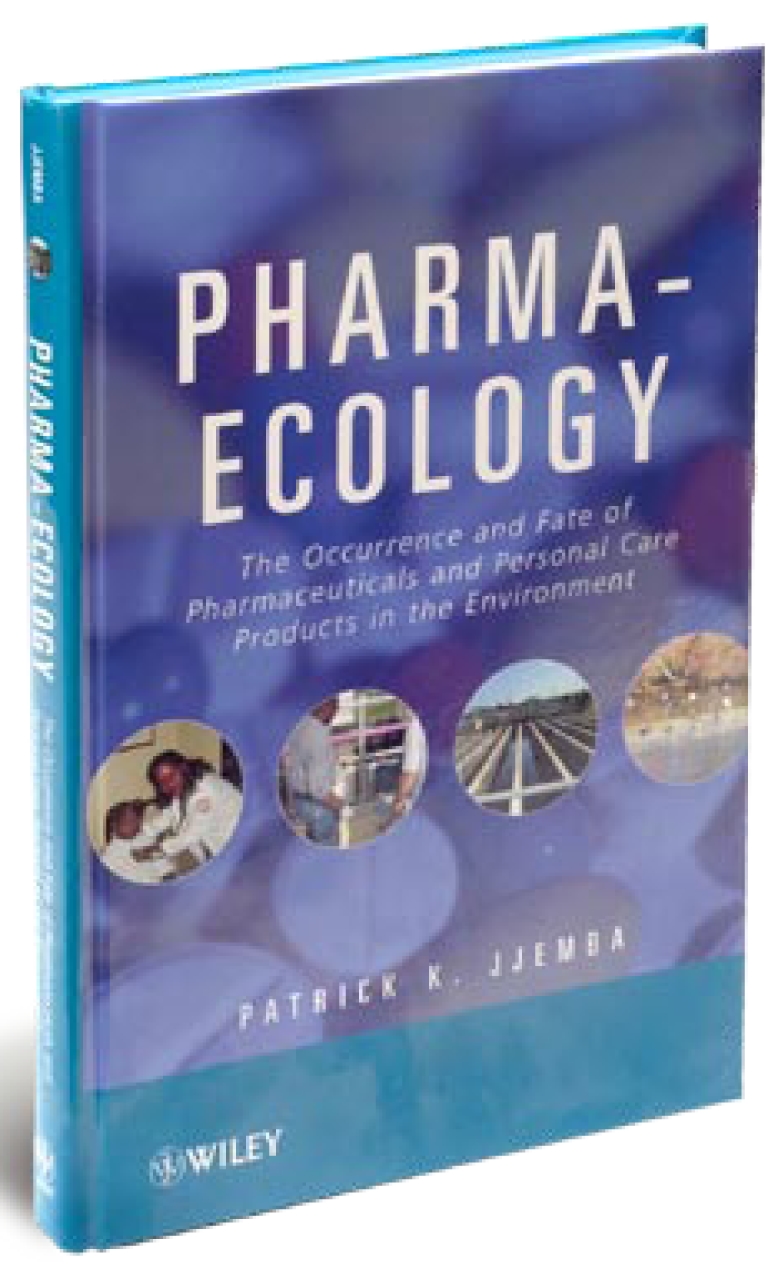# Pharma-Ecology – The Occurrence and Fate of Pharmaceuticals and Personal Care Products in the Environment

**Published:** 2009-04

**Authors:** Rolf U. Halden

**Affiliations:** Rolf U. Halden is associate professor of civil, environmental, and sustainable engineering at Arizona State University and adjunct associate professor of environmental health sciences at Johns Hopkins University. He studies environmental exposures to PPCPs and has spoken on this issue to the U.S. Environmental Protection Agency, Food and Drug Administration, and the National Academies

Pharmaceuticals and personal care products (PPCPs) in the environment are a hot topic. Emitted from various sources, veterinary antibiotics, prescription drugs, and beauty products continuously enter the environment, where they have been detected at trace levels in reclaimed wastewater, surface and ground water, sludge-amended agricultural soils, aquatic and terrestrial biota, as well as in raw and finished drinking water. Fueled by media coverage, the general public has taken notice and is challenging the scientific and regulatory community to assess potential human health and ecological risks, and to take appropriate action where needed. Patrick K. Jjemba, a senior research analyst for innovation and technology with American Water, takes on the ambitious task of framing the complex issue of PPCPs in the environment.

The first chapter introduces industrial agriculture, domestic waste-water treatment, and the land application of municipal sewage sludge (biosolids) as principal sources of PPCPs in the environment. For balance, usage rates of veterinary antibiotics are provided from both the agricultural industry and concerned scientists. Personal care products are categorized as fragrances and musks, detergents, and disinfectants. Human pharmaceuticals are discussed based on the ailment targeted. Jjemba suggests that the increase in the use of antidepressants is attributable at least partly to aggressive marketing. This treatise is comprehensive and well referenced.

The next, key chapter deals with the detection and occurrence of PPCPs in the environment. The brief description of instrumentation contains minor technical inaccuracies. The challenge of accurately determining environmental concentrations in the parts-per-trillion (ppt) range is correctly portrayed, but readers receive no guidance on how to distinguish high-quality data from less rigorous monitoring information. A table presenting concentrations of individual compounds across different media to illustrate the wide range of environmental occurrences, from ppt levels in surface and drinking water to parts-per-million levels (ppm) in sludge-amended soils would have been useful. Although most information is presented adequately, readability is diminished by a missing figure caption, conflicting figure labels, environmental transport data from multiple independent studies that should not be interpreted as a single data set, a cited but unreferenced study, and some typos in reproduced equations.

Ensuing chapters on ecopharmacokinetics, pharmacodynamics, and ecotoxicity are detailed and much more convincing. These important aspects make the book a worthwhile investment, and also help distinguish it from other PPCP compendia. A valuable chapter on technologies for wastewater and water treatment provides a good overview of conventional and advanced treatment technologies and their effectiveness in removing the trace contaminants of interest. Missing from this section are pollution prevention strategies, such as separation of urine and feces at the source followed by individual treatment of both waste streams, as well as alternatives to centralized sanitary sewer systems, such as no-flush composting toilets.

The final pages are dedicated to future needs, including the development of better risk assessment approaches, a consideration of PPCPs as mixtures rather than single compounds, the potential of green chemistry in drug design, the utility of quantitative structure activity relationships in ecotoxicology, and the use of genomic approaches in bioassays. Jjemba closes with a call for a reexamination of the drug approval process and for tighter control of patient-directed advertising by the pharmaceutical industry.

How is this book different from others on the topic? Jjemba is the sole author, providing comprehensive coverage of the various PPCP aspects in a logical order and in a consistent style of presentation. Edited compendia containting contributions form multiple authors can be more authoritative but also more disjointed. Jjemba acknowledges the risk that some readers may be “dissatisfied with the level of coverage of one aspect or another, particularly aspects that directly relate to their respective discipline.”

Jjemba’s likely audience is newcomers seeking a general understanding of the subject and experts interested in broadening their perspectives. The more than 600 references provided will help readers pursue original sources.

## Figures and Tables

**Figure:**